# Ibuprofen Versus Indomethacin for Medical Closure of the Patent Arterial Duct: A Pooled Analysis by Route of Administration

**DOI:** 10.7759/cureus.274

**Published:** 2015-06-04

**Authors:** Rohit Loomba, Karan Nijhawan

**Affiliations:** 1 Cardiology Dept., Children's Hospital of Wisconsin; 2 Medicine, Rush University Medical Center

**Keywords:** patent arterial duct, patent ductus arteriosus, ibuprofen, ligation, premature, indomethacin

## Abstract

Introduction:

Preterm infants are at increased risk of having a patent arterial duct (PAD). PADs may cause congestive heart failure, respiratory distress, necrotizing enterocolitis, and renal impairment. Consequently, in some infants, it becomes necessary to attempt closure of the PAD. Surgical closure can be difficult in small infants and is not without its risks; thus, medical closure offers advantages. Cyclooxygenase inhibitors have been used for medical closure of the PAD with both ibuprofen and indomethacin having been used clinically.

Methods:

We performed a systematic review of the literature to identify all studies comparing ibuprofen and indomethacin. Studies comparing ibuprofen and indomethacin for closure of the PAD in premature infants were included in the meta-analysis. A subanalysis was performed to compare the route of administration. Efficacy endpoints studied were PAD closure and surgical ligation while adverse effects studied were death in the first month of life, necrotizing enterocolitis, gastrointestinal bleeding, intestinal perforation, bronchopulmonary dysplasia in the first month of life, Grade 3 or 4 intraventricular hemorrhage, and change in the serum creatinine after treatment.

Results:

Ibuprofen and indomethacin were equally effective in closing the PAD in premature infants and demonstrated no difference in the incidence of adverse events. In respect to the route of administration, oral ibuprofen was as effective as intravenous indomethacin. When comparing both drugs via the intravenous route, the only difference noted between the ibuprofen and indomethacin was that ibuprofen was associated with a lesser increase in serum creatinine after treatment.

Conclusion:

Ibuprofen and indomethacin are equally effective in PAD closure without any difference in the incidence of adverse events. Importantly, oral ibuprofen was as effective as intravenous indomethacin.

## Introduction

The arterial duct (AD) is a vascular structure that is necessary for fetal life, allowing for much of the right ventricular output to be shunted from the pulmonary artery to the descending aorta, thereby allowing for the developing lungs to receive enough blood to sustain development. Oxygenation, however, occurs at the level of the placenta during fetal life, and thus, blood does not need to pass through the pulmonary vasculature itself. The AD closes shortly after birth. In infants born at 30 to 40 weeks gestational age, the AD closes by 72 hours in 90%, and in infants born at greater than 40 weeks gestational age, the AD closes in 100% by 72 hours [1-3].

The AD is considered to be a patent ductus arteriosus (PAD) if it does not close by 72 hours of life. The prevalence of PAD is not entirely known but is present in approximately 0.06% of all live births with estimates of silent PADs having a prevalence of 0.1 to 0.2%. The prevalence of PADs is greater in those with lower birth weight and preterm gestation. For infants weighing 1,000 to 1,500 grams, 25% will have a PAD, 70% of whom will require treatment, and for infants less than 1,000 grams, 65% will have a PAD, 85% of whom will require treatment [4-7].

What justifies treatment is currently under debate with many advocating for less treatment to avoid potential adverse effects of either surgical ligation or medical therapy. Others advocate being aggressive with treatment to avoid potential adverse effects of the PAD [8-9].

If the decision is made to treat, medical therapy is an option for premature infants. Medical therapy used to consist of indomethacin; however, over the last few years, ibuprofen has become an increasingly popular alternative. Both are still used in various settings without a common practice. We present a pooled analysis of the efficacy and safety of ibuprofen and indomethacin for the closure of the PAD in preterm infants.

## Materials and methods

A systematic review of the literature was performed to identify manuscripts describing comparisons between ibuprofen and indomethacin for closure of the PAD. This was a newly conducted review, and no previous review protocol has been established for the effectiveness in closing the AD and the need for surgical ligation. The following adverse events were studied: death in the first month of life, necrotizing enterocolitis, gastrointestinal bleeding, intestinal perforation, bronchopulmonary dysplasia in the first month of life, Grade 3 or 4 intraventricular hemorrhage, and change in serum creatinine after attempted treatment.

### Manuscript search and identification strategy

Manuscripts were identified using electronic databases, including PubMed, EMBASE, and Ovid, which were queried using either “ibuprofen” and/or “indomethacin” in combination with “ductus arteriosus” or “arterial duct”. No specific restriction on year of publication was used. Studies in languages other than English were excluded. Resulting studies were then screened by title and abstract with manuscripts suspected of being relevant being retrieved in their entirety. References of these were hand searched for additional relevant manuscripts. No direct contact with manuscript authors was required to obtain full text manuscripts. Only studies with an ibuprofen and indomethacin group were included. Those with no data reported for any of the endpoints of interest or studies with data not deemed suitable for extraction were also excluded.

These full text manuscripts were then reviewed by two of the authors (RL, KN) and assessed for quality. Any disparities in scoring of manuscripts were then discussed and resolved. The Cochrane Handbook for Systematic Review of Interventions was used for quality evaluation. Published manuscripts available in full text were included in this review if they presented data comparing ibuprofen and indomethacin with respect to the outcomes listed above. Studies were included in this analysis if they included at least one of the outcomes identified above.

### Data extraction

Data regarding baseline patient characteristics and identified outcomes were extracted from the manuscripts identified for inclusion. Trial level data were extracted independently with the use of a data collection form by two authors. The data extraction was then independently reviewed by another author to ensure the integrity of the resulting data. Authors of included studies were not contacted for additional data.

### Bias analysis

Bias was assessed using the Cochrane Risk of Bias Tool. Specifically, patient eligibility, randomization and concealment of allocation, blinding, and completeness of outcome data were assessed using this scale. Publication bias was assessed qualitatively using forest plots for endpoints with 10 or more studies included.

### Data analysis

Numeric data are presented as means with standard deviations. Categorical data are presented as frequencies with absolute numbers as well as percentages. Results are presented as pooled odds ratios (OR) with 95% confidence intervals (CI) or as mean difference (MD) where appropriate. Heterogeneity between studies was identified using chi-square and I^2 ^tests. For outcomes with no significant heterogeneity present, a fixed effects model was used. A random effects model was used if either the p-value was significant or the I^2^ statistics was greater than 50%. P-values of ≤ 0.05 were considered statistically significant. The Mantel-Haenszel method was used for dichotomous endpoints while mean difference was used for continuous endpoints. Meta-analysis and forest plot creation were done using RevMan 5.0 (Cochrane Collaboration, Oxford, UK).

Analysis was done separately by route of administration. All endpoints were analyzed in the comparison of intravenous (IV) ibuprofen and IV indomethacin, oral ibuprofen versus IV indomethacin, and oral ibuprofen versus oral indomethacin. Data for all endpoints was not available for all routes of administration.

### Sensitivity analyses

Sensitivity analyses were conducted to determine the effect of study size, study weight, year of publication, and study design.

## Results

### Search results

**Figure 1:**
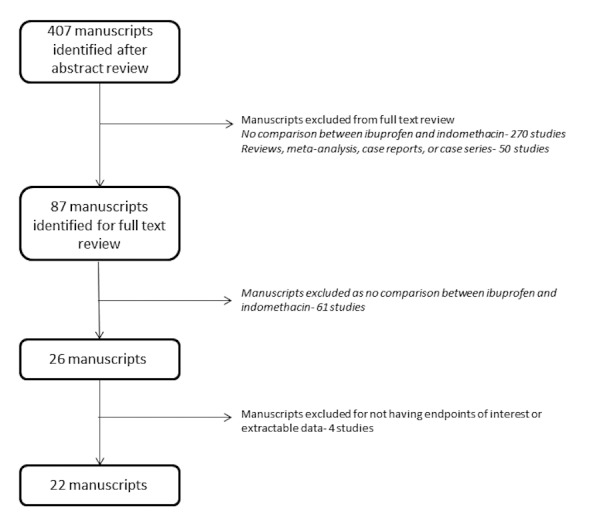
Study methodology Study methodology

A total of 723 manuscripts were identified with 407 remaining after duplicates were identified and removed. Titles and abstracts of these manuscripts were reviewed, and 87 manuscripts were identified for full-text review. Of the 320 manuscripts not advancing to full-text review, 270 were excluded as there was no comparison between ibuprofen and indomethacin and 50 were excluded because they were reviews, meta-analyses, case reports, series, or letters. After a full-text review of the 87 identified manuscripts, 26 studies were identified as comparing the use of ibuprofen and indomethacin for closure of the PAD in preterm infants. Four of these studies contained data for none of the predefined endpoints of interest or had data not suitable for extraction, and thus, a total of 22 studies were included in the final pooled analysis (Figure 1) [10-31]. Of the included studies, 14 (64%) were randomized while eight (36%) were retrospective studies (Table 1).

**Table 1:** Study characteristics Study characteristics

Study	Design	Ibuprofen					Indomethacin				
		n	Male	Gestational age (wks)	Birth weight (kg)	Route	n	Male	Gestational age	Birth weight	Route
*Aly et al*	Randomized	12	8 (67)	32.9 ± 1.6	1.9 ± 0.5	oral	9	4 (44)	31.2 ± 2.5	1.5 ± 0.4	IV
*Chan et al*	Retrospective	43	20 (47)	28.8 ± 2.9	1.1 ± 0.4	IV	52	28 (54)	28.4 ± 3.2	1.1 ± 0.5	IV
*Fanos et al*	Retrospective	20	14 (70)	26.3 ± 2.4	0.9 ± 0.3	IV	20	12 (60)	26.5 ± 2.2	0.9 ±0.2	IV
*Hammerman et al*	Randomized	32	14 (44)	27.8 ± 2.6	1.1 ± 0.4	IV	31	18 (58)	27.8 ± 2.8	1.1 ± 0.5	IV
*Heo et al*	Retrospective	22	9 (41)	32.0 ± 4.0	1.7 ± 0.7	oral	27	13 (41)	31.0 ± 3.4	1.6 ± 0.6	IV
*Katakam et al*	Retrospective	57	--	26.3 ± 2.0	1.0 ± 0.3	IV	65	--	27.3 ± 3.3	1.1 ± 0.5	IV
*Lago et al*	Randomized	94	50 (53)	28.0 ± 2.0	1.1 ± 0.4	IV	81	44 (54)	29.0 ± 3.0	1.2 ± 0.4	IV
*Lee et al*	Retrospective	52	29 (56)	28.8 ± 2.3	1.1 ± 0.2	oral	88	43 (49)	28.9 ± 2.2	1.1 ± 0.2	IV
*Linder et al*	Retrospective	73	32 (44)	27.7 ± 2.5	1.0 ± 0.3	IV	46	22 (48)	27.1 ± 2.8	0.9 ± 0.3	IV
*Mosca et al*	Randomized	8	4 (50)	29.0 ± 1.2	1.0 ± 0.3	IV	8	5 (63)	27.8 ± 1.5	0.9 ± 0.2	IV
*Patel et al*	Randomized	12	--	--	--	IV	6	--	--	--	IV
*Patel et al*	Randomized	18	9 (50)	27.7 ± 3.4	1.2 ± 0.7	IV	15	7 (47)	26.7 ± 2.0	0.9 ± 0.3	IV
*Pezzati et al*	Randomized	9	--	29.1 ± 2.1	1.2 ± 0.4	IV	8	--	29.5 ± 2.6	1.3 ± 0.4	IV
*Pourarian et al*	Randomized	10	7 (70)	31.3 ± 4.4	1.9 ± 0.4	oral	10	6 (60)	33.2 ± 3.1	1.7 ± 0.6	oral
*Sivanandan et al*	Retrospective	70	44 (63)	27.0 ± 2.1	1.0 ± 0.3	oral	54	27 (50)	27.0 ± 2.3	1.0 ± 0.3	IV
*Su et al*	Randomized	60	34 (57)	25.3 ± 1.5	0.8 ± 0.1	IV	59	33 (56)	25.3 ± 1.5	0.8 ± 0.1	IV
*Su et al*	Randomized	32	--	--	--	IV	31	--	--	--	IV
*Van Overmeire et al*	Randomized	20	--	29.0 ± 2.4	1.3 ± 0.5	IV	20	--	28.7 ± 1.9	1.2 ± 0.4	IV
*Van Overmeire et al*	Randomized	74	--	29.0 ± 2.3	1.2 ± 0.4	IV	74	--	29.0 ± 2.1	1.2 ± 0.4	IV
*Yadav et al*	Randomized	48	26 (54)	29.7 ± 3.2	1.4 ± 0.5	oral	35	17 (49)	30.3 ± 3.1	1.4 ± 0.5	oral
*Yang et al*	Retrospective	22	9 (41)	26.7 ± 1.0	0.8 ± 0.1	oral	26	6 (23)	27.0 ± 1.3	0.9 ± 0.1	IV
*Chotigeat et al*	Randomized	15	8 (53)	30.8 ± 2.3	1.4 ± 0.4	oral	15	7 (48)	29.9 ± 2.9	1.4 ± 0.4	IV

### Patient characteristics

A total of 1,583 patients were included in this pooled analysis. Of the 22 studies, 14 compared IV ibuprofen to IV indomethacin with 552 patients in the ibuprofen group and 516 in the indomethacin group; six studies compared oral ibuprofen to IV indomethacin with 193 patients in the ibuprofen group and 219 in the indomethacin group; and two studies compared oral ibuprofen to oral indomethacin with 58 patients in the ibuprofen group and 45 in the indomethacin group. Additional data regarding patient characteristics can be found in Table 1.

### PAD closure

**Figure 2:**
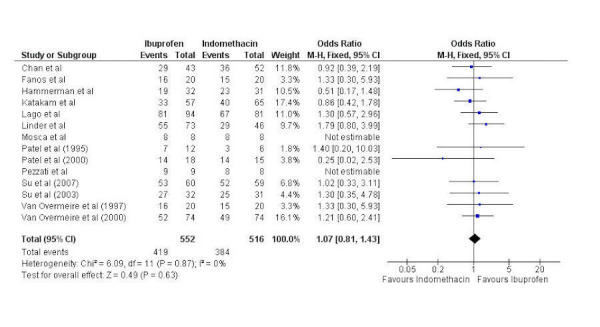
Forest plot Forest plot comparing PAD closure between intravenous ibuprofen and intravenous indomethacin

Fourteen studies were pooled comparing PAD closure with IV ibuprofen and IV indomethacin with a total of 552 patients having received ibuprofen and 16 having received indomethacin. Significant heterogeneity was not present (chi-squared=6.09, p=0.87, I^2^=0%) so a fixed-effects model was used. There was no statistically significant difference in PAD closure with IV ibuprofen when compared to IV indomethacin (odds ratio 1.07, 95% confidence interval 0.81 to 1.43) (Figure 2).

**Figure 3:**
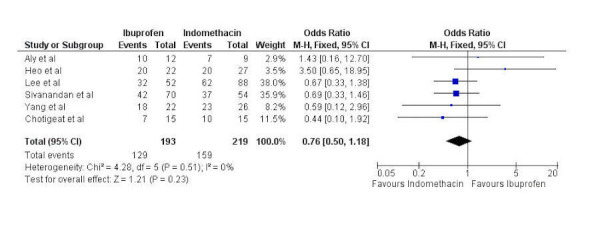
Forest plot Forest plot comparing PAD closure between oral ibuprofen and intravenous indomethacin

Six studies were pooled comparing PAD closure with oral ibuprofen and IV indomethacin with 193 patients having received ibuprofen and 219 having received indomethacin. A fixed-effects model was used as significant heterogeneity was not present (chi-squared=0.76, p=0.51, I^2^=0%). There was no statistically significant difference in PAD closure with oral ibuprofen when compared to IV indomethacin (odds ratio 0.76, 95% confidence interval 0.50 to 1.18) (Figure 3).

**Figure 4:**
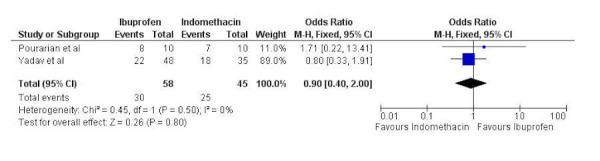
Forest plot Forest plot comparing PAD closure between oral ibuprofen and oral indomethacin

Two studies contained data comparing PAD closure with oral ibuprofen and oral indomethacin with 58 patients in the ibuprofen group and 45 in the indomethacin group. Significant heterogeneity was not present (chi-squared=0.45, p=0.50, I^2^=0%) so a fixed-effects model was used. There was no statistically significant difference between oral ibuprofen and oral indomethacin in respect to PAD closure (odds ratio 0.90, 95% confidence interval 0.40 to 2.00) (Figure 4).

### Need for surgical ligation

Data regarding the need for surgical ligation comparing IV ibuprofen and IV indomethacin was available from nine studies with 418 patients having received ibuprofen and 417 having received indomethacin. A fixed-effects model was used as there was no significant heterogeneity (chi-squared 3.69, p=0.88, I^2^=0%). No statistically significant difference was noted in the need for surgical ligation between IV ibuprofen and IV indomethacin (odds ratio 0.86, 95% confidence interval 0.58 to 1.28).

Four studies compared need for surgical ligation in 159 patients having received oral ibuprofen and 183 having received IV indomethacin. A fixed-effects model was used as there was not significant heterogeneity (chi-squared=2.47, p=0.48, I^2^=0%). The need for surgical ligation did not statistically significantly differ between oral ibuprofen and IV indomethacin (odds ratio 0.78, 95% confidence interval 0.45 to 1.37).

### Death in first month of life

A total of nine studies compared death in the first 30 days of life between 485 patients having received IV ibuprofen and 459 having received IV indomethacin. Significant heterogeneity was not present (chi-squared=11.24, p=0.19, I^2^= 29%). There was no statistically significant difference between those having received IV indomethacin and those having received IV ibuprofen (odds ratio 1.03, 95% confidence interval 0.67 to 1.58).

### Necrotizing enterocolitis

Eight studies compared the occurrence of necrotizing enterocolitis between IV ibuprofen and IV indomethacin with 412 and 413 patients in the two groups, respectively. No significant heterogeneity was present so a fixed-effects model was used (chi-squared=4.40, p=0.73, I^2^=0%). No statistically significant difference in necrotizing enterocolitis was noted between IV ibuprofen and IV indomethacin (odds ratio 0.97, 95% confidence interval 0.63 to 1.50).

Data regarding necrotizing enterocolitis comparing oral ibuprofen and IV indomethacin was available from five studies with 181 patients having received ibuprofen and 210 having received indomethacin. A fixed effects model was used as there was no significant heterogeneity (chi-squared=3.93, p=0.42, I^2^=0). No statistically significant difference was noted in necrotizing enterocolitis between oral ibuprofen and IV indomethacin (odds ratio 0.60, 9% confidence interval 0.30 to 1.24).

### Gastrointestinal bleeding

Four studies compared the occurrence of gastrointestinal bleeding between 155 patients having received IV ibuprofen and 162 patients having received IV indomethacin. Significant heterogeneity was not present so a fixed-effects model was utilized. No statistically significant difference was noted in gastrointestinal bleeding between IV ibuprofen and IV indomethacin (odds ratio 1.40, 95% confidence interval 0.73 to 2.69).

A total of three studies compared gastrointestinal bleeding between oral ibuprofen and IV indomethacin with 144 and 169 patients in each group, respectively. A fixed-effects model was used as significant heterogeneity was not present (chi-squared=2.75, p=0.25, I^2^=27%). A statistically significant difference in gastrointestinal bleeding was not found between oral ibuprofen and IV indomethacin (odds ratio 0.62, 95% confidence interval 0.31 to 1.27).

### Intestinal perforation

Seven studies compared intestinal perforation in 380 patients having received IV ibuprofen and 382 patients having received IV indomethacin. Significant heterogeneity was not present so a fixed-effects model was used (chi-squared=3.09, p=0.80, I^2^=0%). A statistically significant difference in intestinal perforation was not noted between IV ibuprofen and IV indomethacin (odds ratio 1.09, 95% confidence interval 0.54 to 2.20).

### Bronchopulmonary dysplasia in the first month of life

Data regarding the frequency of bronchopulmonary dysplasia in the first month of life was pooled from six studies with 323 patients having received IV ibuprofen and 317 having received IV indomethacin. A fixed-effects model was used as there was no significant heterogeneity noted (chi-squared=5.69, p=0.34, I^2^=12%). No statistically significant difference was noted in bronchopulmonary dysplasia in the first month of life between IV ibuprofen and IV indomethacin (odds ratio 1.09, 95% confidence interval 0.77 to 1.54).

A total of four studies were pooled to compare the frequency of bronchopulmonary dysplasia in the first month of life between 120 patients having received oral ibuprofen and 122 having received IV indomethacin. Significant heterogeneity was not present so a fixed-effects model was used (chi-squared=3.35, p=0.34, I^2^=10%). No statistically significant difference in the frequency of bronchopulmonary dysplasia in the first month of life was noted between oral ibuprofen and IV indomethacin (odds ratio 0.80, 95% confidence interval 0.47 to 1.36).

### Grade 3 or 4 intraventricular hemorrhage

Seven studies were pooled to compare the frequency of Grade 3 or 4 intraventricular hemorrhage between 392 patients having received IV ibuprofen and 393 patients having received IV indomethacin. A fixed-effects model was used as no significant heterogeneity was noted (chi-squared=5.66, p=0.46, I^2^=0%). No significant difference was noted in Grade 3 or 4 intraventricular hemorrhage between IV ibuprofen and IV indomethacin (odds ratio 0.79, 95% confidence interval 0.47 to 1.31).

### Change in creatinine

**Figure 5:**
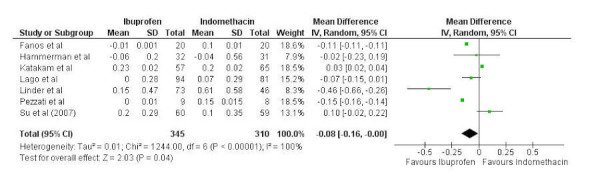
Forest plot Forest plot comparing change in creatinine between intravenous ibuprofen and intravenous indomethacin

A total of seven studies were pooled to compare changes in serum creatinine after attempted treatment between 345 patients having received IV ibuprofen and 310 patients having received IV indomethacin. Significant heterogeneity was present so a random-effects model was fixed (chi-squared=1244.00, p<0.001, I^2^=100%). A statistically significant difference in change in creatinine was present and favored ibuprofen (mean difference -0.08, 95% confidence interval -0.16 to 0.00) (Figure 5).

**Figure 6:**
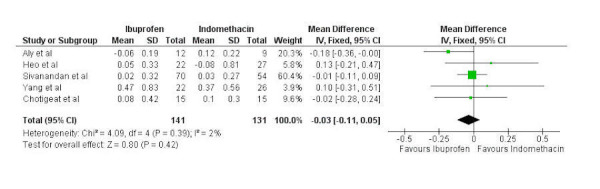
Forest plot Forest plot comparing change in creatinine between oral ibuprofen and intravenous indomethacin

Five studies were pooled to compare changes in creatinine between 141 patients having received oral ibuprofen and 131 patients having received IV indomethacin. A fixed-effects model was used as no significant heterogeneity was present (chi-squared=4.09, p=0.39, I^2^=2%). No significant difference was noted between oral ibuprofen and IV indomethacin in regards to change in creatinine (mean difference -0.03, 95% confidence interval -0.11 to 0.05) (Figure 6).

**Figure 7:**
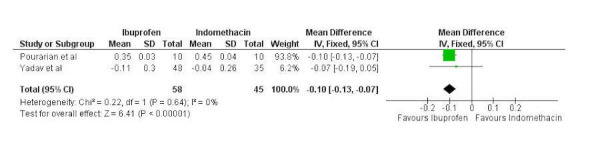
Forest plot Forest plot comparing change in creatinine between oral ibuprofen and oral indomethacin

Data regarding change in creatinine between oral ibuprofen and oral indomethacin was available from two studies with 58 and 45 patients in the groups, respectively. A fixed-effects model was used as there was no significant heterogeneity present (chi-squared=0.22, p=0.64, I^2^=0%). A statistically significant difference in change in creatinine was present, favoring oral ibuprofen (mean difference -0.10, 95% confidence interval -0.13 to -0.07) (Figure 7).

## Discussion

This pooled analysis of 22 studies with 1,582 patients demonstrates that both ibuprofen and indomethacin are effective for PAD closure. Route of administration does not seem to affect efficacy and even oral ibuprofen seems to be equally effective as IV indomethacin. There was no difference in the incidence of surgical ligation between those given indomethacin and those given ibuprofen.

In regards to safety, there was no difference in the incidence of adverse outcomes, such as death in the first month of life, necrotizing enterocolitis, gastrointestinal bleeding, intestinal perforation, bronchopulmonary dysplasia in the first month of life, and Grade 3 or 4 intraventricular hemorrhage. There was a smaller increase in serum creatinine after attempted treatment associated with IV ibuprofen when compared to IV indomethacin and when oral ibuprofen was compared to IV indomethacin.

Previous studies have demonstrated a similar difference between ibuprofen and indomethacin in regards to change in creatinine [12, 18, 22]. Other studies have demonstrated that ibuprofen has less potential of adverse effects on renal function when compared to indomethacin using other endpoints, such as urine output as well as oliguria, which were not included in the current analysis due to the relatively low patient number in comparison to other endpoints. Urine output has been demonstrated to be higher in infants receiving ibuprofen rather than indomethacin for PAD closure.

The AD consists of an outer medial layer that consists of circumferential smooth muscle fibers supplied by thin-walled vasa vasorum while the inner medial layer is avascular and consists of longitudinal smooth muscle fibers. The internal elastic lamina separates this medial layer from the intima which is thin until the final end of gestation at which point it thickens. This is in contrast to the main pulmonary artery and the aorta which are comprised of circumferential elastic fibers [32].

The AD facilitates the passage of approximately 30% to 45% of the combined ventricular output in the human fetus, allowing for much of the blood to bypass the fetal lungs, which are immature and fluid filled, thus allowing them to complete development without the burden of a large amount of blood flow [33-35]. Since gas exchange occurs in the placenta, the fetal lungs require blood only for their own oxygenation and, therefore, do not require the entirety of the right ventricular output. The proportion of cardiac output that traverses the AD may presumably vary based on the amount of blood that is shunting at the atrial level via the foramen ovale, with the increased atrial level shunting leading to a decreased proportion of blood flow through the AD. It has been demonstrated that blood with a higher oxygen content in the inferior vena cava has a higher flow velocity, which facilitates streaming of this blood preferentially through the foramen ovale. This then allows for more oxygenated blood to reach the left ventricle to be pumped to the coronary arteries and head and neck vessels via the ascending aorta. While this occurs, less oxygenated blood streams preferentially into the right ventricle and into the AD or fetal lungs [36-38].

While the specific mechanisms that mediate fetal patency of the AD are not entirely elucidated, several have been proposed, which include low partial pressure of oxygen, maternal prostaglandins, adenosine, and nitric oxide production [39-46]. Since these factors promote AD patency, it thus becomes intuitive that the converse of these would result in the closure of the ductus arteriosus which in fact is seen postnatally. The AD remains open in humans for approximately 24 to 48 hours after birth and will often have bi-directional flow initially, followed by a left to right shunt before it closes. In the immediate postnatal period when pulmonary vascular resistance is still elevated, a systolic shunt from the pulmonary artery to the descending aorta is present while, in diastole, a shunt from the aorta to the pulmonary artery is present. Once pulmonary vascular resistance has decreased further, the shunt becomes purely left to right. While the AD is closing, it is still reactive to certain mediators, such as the pressure of oxygen, and this decrease in the partial pressure of oxygen will lead to potential widening of the AD and an increase in pulmonary vascular resistance with the potential return of right to left shunting. Closure of the AD is found in 44% of full-term infants by 24 hours, 88% by 48 hours, and 100% by 72 hours [47].

Thickening of the intimal layer and the resulting decrease in luminal diameter facilitates closure of the AD. While this thickening occurs throughout gestation, it is particularly marked after birth. The internal elastic lamina also begins to fragment and allow smooth muscle and endothelial cell proliferation. Eventually, the AD constricts to a point where hypoxia develops. The vasa vasorum provides blood to the adventitia while the intima and the media receive blood directly from the lumen. Thus, hypoxia ensues as the AD constricts decreases flow to the media. With this hypoxia comes apoptosis and eventual fibrous tissue deposition, which completes the AD closure. In the former phases of this process where smooth muscle constriction and intimal thickening is occurring, the process may be reversible. Once the fibrous deposition has occurred, however, the process is no longer reversible [48].

Oxygen clearly plays a role in AD closure. Studies have demonstrated that increased partial pressure of oxygen resulted in AD closure [49-50]. This, however, only appears to be the response in late gestation as even exceedingly high partial pressures of oxygen do not induce constriction of the AD at earlier gestation. The mechanism behind this is unclear as AD constriction does occur with other agents, such as calcium and acetylcholine [50]. Studies demonstrating the need for an intact cytochrome oxiADse system for oxygen to produce AD constriction have raised the possibility that perhaps oxygen directly causes smooth muscle contraction. It has been hypothesized that perhaps oxygen binding to the cytochrome p450 hemoprotein may lead to membrane depolarization with calcium influx, which then leads to muscle contraction. It has also been suggested that perhaps oxygen-sensitive potassium channels in smooth muscle may cause depolarization and activation of voltage-sensitive calcium channels with subsequent smooth muscle contraction [51-52]. Other studies have also implicated the Rho-kinase system as well as endothelin-1 release as mechanisms by which oxygen may play a role in AD closure [53-54]. Additionally, the partial pressure of oxygen required to induce constriction has also been noted to decrease with increasing gestational age [50].

Nitric oxide, a potent vasodilator, is produced by endothelial cells utilizing the nitric oxide synthase. Nitric oxide synthase has been found in the AD and its surrounding endothelial tissue. Specific nitric oxide synthase inhibitors have also been demonstrated to be ineffective in antagonism of this system in the AD at low partial pressures of oxygen, which may also explain the mechanism by which oxygen affects AD constriction. Additionally, this system thus facilitates AD closure at a later gestational age [44].

Other vasoactive agents, such as adenosine, acetylcholine, bradykinin, and norepinephrine, have also been demonstrated to lead to AD constriction and have synergistic effects to the AD’s response to oxygen. The absence of any of these agents, however, does not impair oxygen-mediated AD constriction. The precise role of these agents is not clearly defined [46, 55].

Of particular importance is the effect of prostaglandins on AD closure as both ibuprofen and indomethacin target the prostaglandin pathway. Cyclooxygenase is the enzyme responsible for synthesizing prostaglandins from arachidonic acid [56]. There are two varieties of this enzyme and both have been found to participate in prostaglandin synthesis in the wall of the AD, resulting in both prostaglandin E_2_ and prostaglandin I_2_. While prostaglandin I_2 _is more abundant, prostaglandin E_2 _is more potent with the AD being nearly 1,000 times more sensitive to prostaglandin E_2_, which is believed to play a vital role in maintaining the AD during fetal life [39]. In fact, prostaglandin E_2 _levels are higher in the fetus than in the mother with levels falling rapidly after birth, such that adult levels are reached by three hours of life [42]. What is particularly intriguing is that reductions in pulmonary blood flow, as may be seen secondary to lung disease or pulmonary vasoconstriction, can delay this fall in prostaglandin E_2 _levels since the lungs are the site of its metabolism. The partial pressure of oxygen does not impact the effect of prostaglandin E_2 _on AD constriction. Premature infants have both continued production of prostaglandin E_2 _but also an increased response to it [57].

These findings regarding the effect of prostaglandins on AD closure have important clinical implications. Both ibuprofen and indomethacin are nonselective cyclooxygenase inhibitors and also inhibit the synthesis of prostaglandin E_2_. Thus, understanding of the previously mentioned characteristics of the AD make the use of cyclooxygenase inhibitors intuitive in the setting of premature infants with PAD [58].

This study demonstrates that ibuprofen and indomethacin are equally effective in closing the PAD with ibuprofen having a renoprotective effect with some dosage routes. This current manuscript also reviews the embryology and physiology of the AD. This analysis, however, is not without its limitations. Pooled data is study-level rather than patient-level, thus limiting the opportunity for specific meta-regression. Additionally, some endpoints had significant heterogeneity. Even those endpoints without quantitative endpoints may have qualitative heterogeneity. Additionally, no cost analysis could be conducted. There was a small number of studies available to pool in regards to the comparison of the oral ibuprofen and oral indomethacin as well.

## Conclusions

Ibuprofen and indomethacin are both equally effective in closure of the PAD in preterm infants. Adverse effects associated were not found to differ between the two, although ibuprofen was associated with a lower increase in creatinine.
